# Disseminated hyaline ring granuloma in the omentum of a dog

**DOI:** 10.1186/s13028-017-0294-z

**Published:** 2017-04-28

**Authors:** Izabella Dolka, Anna Gruk-Jurka, Piotr Jurka, Beata Dolka, Joanna Bonecka

**Affiliations:** 1 Department of Pathology and Veterinary Diagnostics, Faculty of Veterinary Medicine, Warsaw University of Life Sciences, Nowoursynowska 159c, 02-776 Warsaw, Poland; 2 Department of Small Animal Diseases with Clinic, Faculty of Veterinary Medicine, Warsaw University of Life Sciences, Nowoursynowska 159c, 02-776 Warsaw, Poland

**Keywords:** Hyaline ring granuloma, Pulse granuloma, Dog, Histopathology

## Abstract

**Background:**

Hyaline ring granuloma (HRG) is an uncommon histopathologic finding of unsolved etiopathogenesis. According to the exogenous theory, HRG develops due to implantation of foreign material, most probably indigestible plant fragments. HRG is a comparatively rare condition in humans, mostly involving the oral cavity with very rare extraoral locations.

**Case presentation:**

An 1-year-old mixed-breed dog in good condition was presented for routine ovariohysterectomy. Disseminated HGR were accidentally found in the omental adipose tissue during surgery. Histopathology revealed the presence of ring-like hyaline structures surrounded by granulomatous inflammation including foreign body-type multinucleated giant cells. The histochemical examinations indicated the exogenous plant origin of the foreign material.

**Conclusions:**

The lesions were similar to the findings in humans with HRG. The definitive diagnosis remains largely based on histopathological examination supported by special histochemical stains. To the best of our knowledge, this is the first case of hyaline ring granuloma reported in a non-human species. Moreover, the omentum is an uncommon location for this condition.

## Background

Hyaline ring granuloma (HRG) is a rare inflammatory lesion with unresolved etiopathogenesis and being characterized histologically by the presence of hyaline rings and foreign-body giant cells [1–3]. The first cases of HRG were reported in humans as a pulmonary inflammatory response resulting from aspiration of vegetable particles [4] and oral inflammatory reaction to particles of food implanted into the mucosa by a denture [5]. Most HRG affect the oral cavity, less common sites include extraoral locations, e.g., the lungs, rectum, gall bladder, urinary bladder, skin and oviduct [2, 6–8]. HRG has been described as a distinct entity under a variety of terms such as giant cell periostitis with hyaline change, giant cell hyaline angiopathy, hyaline bodies (or Rushton bodies), and chronic mandibular periostitis with hyaline rings [2, 9]. Two theories regarding the etiopathogenesis of this unusual lesion have been proposed. The exogenous theory suggests that foreign material, most likely plant food particles, is responsible for the formation of hyaline ring-like structures and induces a chronic granulomatous response. Therefore, the term “pulse granuloma” (PG) derived from the late thirteenth century French words ‘pouls, pols’ and from Latin ‘puls’ meaning ‘thick gruel, porridge, mush’, which referred to the foreign body reaction against the implantation of the seeds of a leguminous pod such as peas, beans and lentils has been proposed [2]. Hence, it was also called food-induced granuloma [1–3, 10]. Most published cases of HRG supports the theory of a plant/vegetable etiology [7]. To elucidate the pathogenesis of HRG, experimental studies in guinea pigs, cats, rats and rabbits have been done [3, 4]. Talacko and Radden [3] reported a progressive development of oral HRG in rats and concluded that breakdown of the plant material implanted into the tissue depended on time and that plant particles were rapidly digested and altered by the host response. The cellulose moiety of the plant fibers persisted in the form of a hyaline material inducing a foreign body reaction. The endogenous theory suggests that hyaline rings derive from hyaline degeneration of the walls of blood vessels or extravasated serum proteins. Hyalinization develops as a consequence of the presence of vascular collagenous fibers (sharing similarities with cellulose) and plasma proteins [11, 12]. Therefore, Dunlap and Barker [11] suggested the term ‘giant cell hyaline angiopathy.’ Hase [13] considered development of oral HRG as being associated with infection by *Candida glabrata* (formerly *Torulopsis glabrata*). As the origin of these structures is still unsolved and it is important to use a uniform nomenclature to avoid controversies in diagnostics, the descriptive term ‘hyaline ring granuloma’ seems most adequate [14]. Non-experimental HRG has not been reported in animals. Here we report a case of omental HRG in a dog.

## Case presentation

**Fig. 1:**
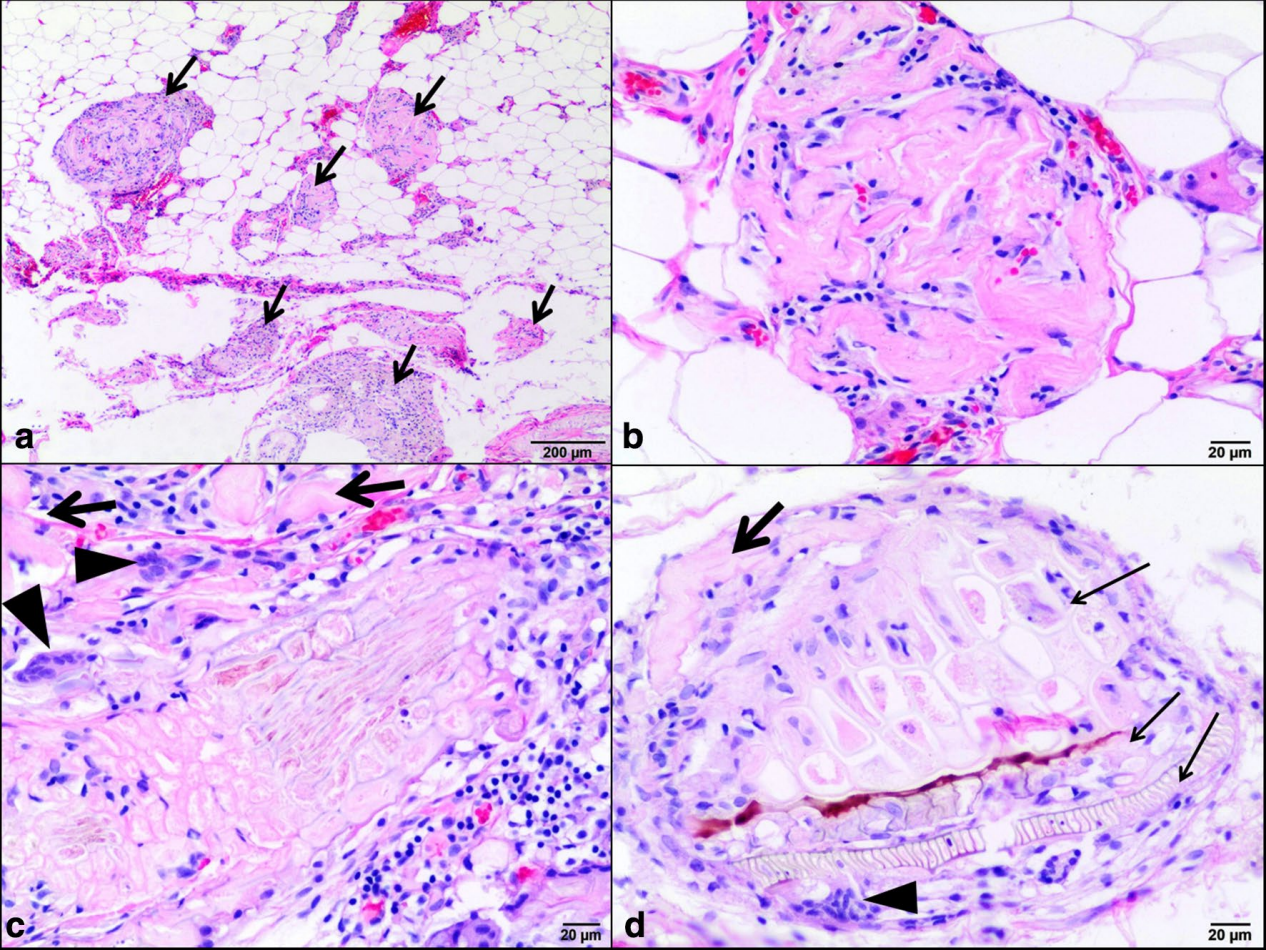
Photomicrographs of hyaline ring granulomas. **a** Small lesions disseminated in the omental adipose tissue. HE, *bar* 200 μm. **b** Corrugated, eosinophilic structures (hyaline rings) associated with inflammatory cell infiltrate entrapment within the rings. HE, *bar* 20 μm. **c** Presence of foreign material, most likely of plant origin, surrounded by hyaline rings (*arrows*) and chronic granulomatous inflammation with multinucleated foreign-body type giant cells reaction (*arrowheads*). HE, *bar* 20 μm. **d** Plant internal structure characterized by the presence of thin-walled cells forming a honeycomb structure suggestive of phloem, and tubular structures resembling tracheids in the xylem with the appearance of microscopic coils (*thin arrows*). Implanted plant particles are associated with hyaline rings (*arrow*) and multinucleated giant cell (*arrowhead*). HE, *bar* 20 μm

**Fig. 2:**
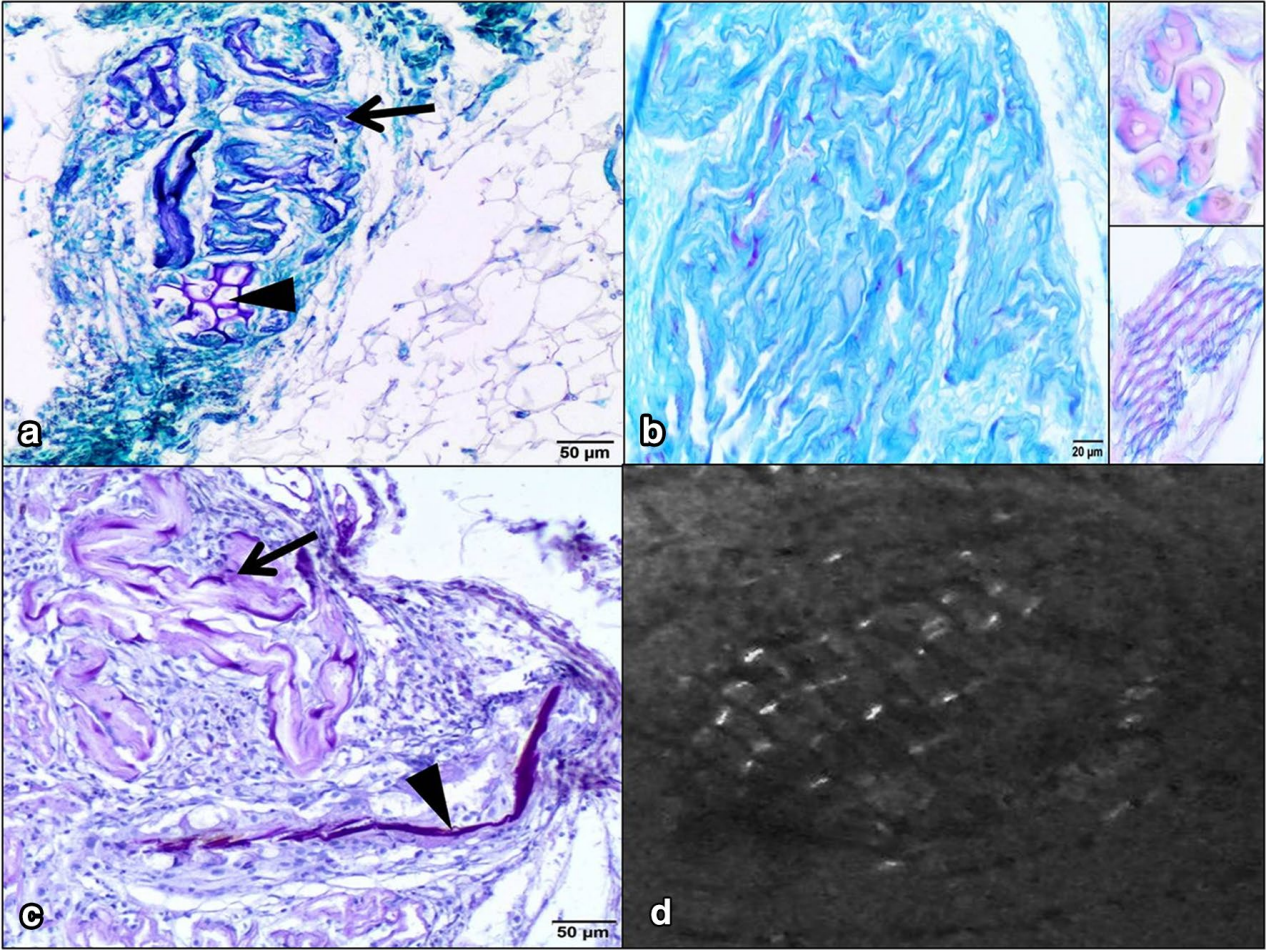
Photomicrographs of sections stained with selected histochemical methods and microscopy in polarized light for the visualization of hyaline ring granulomas in the omental adipose tissue. **a** PAS-positive staining of plant matter (*arrowhead*) and hyaline structures (*arrow*) surrounded by the connective and adipose tissues. PAS, *bar* 50 μm. **b** Most hyaline ring-like structures stained blue with alcian blue. *Bar* 20 μm. *Insets* plant cells stained blue, partially lignified and stained magenta by AB. *Bar* 20 μm. **c** PAS-positive diastase-resistant staining (PASD) of plant material (*arrowhead*) associated with the presence of cellulose and hyaline structures (*arrow*). *Bar* 50 μm. **d** Plant particles showing birefringence under polarized light. The same plant structure is presented in Fig. 1d. *Bar* 20 μm

An approximately 1-year-old mixed-breed female dog was presented at the Small Animal Clinic, Warsaw University of Life Sciences, Poland, for routine ovariohysterectomy. The dog had been adopted and dewormed, and had received its first vaccinations a few months before. On physical examination, the dog was in good condition with a body score of 4 [15] and without any clinical signs apart from a small, firm skin nodule on the chest wall close to the right elbow. This subcutaneous lesion turned out to be a pellet encapsulated by the fibrous tissue. Hematological and biochemical profiles were unremarkable. During ovariohysterectomy, multiple up to 1 mm in diameter white, flat foci scattered on the surface of the greater omentum were present as incidental findings. These punctuate lesions had the appearance of finely dispersed powder talc in the omental adipose tissue and were no longer clearly visible after a 24-h fixation in 10% formalin. Samples of the omentum were submitted for histopathological examination, processed routinely, sectioned and stained with hematoxylin-eosin (HE). Moreover, additional histochemical stains were used: periodic acid-Schiff (PAS), alcian blue at pH 2.5 (AB), PAS with and without predigestion with diastase (PASD), as well as Masson trichrome, Ziehl-Neelsen (ZN) and Congo red stains. Moreover, a microscopic examination under polarized light was done. Chest X-ray and abdominal ultrasound were performed but showed no abnormalities. Histopathology of the omental tissue revealed eosinophilic, convoluted, thin hyaline structures scattered multifocally. Particles of foreign material were found between the ring-like structures in some areas and were surrounded by few multinucleated foreign body giant cells lymphocytes and macrophages (Fig. 1a–d). Some of these particles had a honeycomb or tubular structures with spiral or pitted thickenings suggesting the components of plant tissue such as phloem and tracheids of the xylem. The areas had fibrosis but calcification and metaplastic bone formation were not observed. The plant materials and hyaline ring structures were positive for PAS, AB (with variation in intensity) and PASD (Fig. 2a–c). The sections were negative for Congo red stain (lack of apple-green birefringence under a polarizing microscope) and for acid fast bacilli on ZN staining. The plant materials and hyaline rings were stained very pale green with Masson trichrome compared to deep green staining of collagen fibers in connective tissue around vessels. The plant structures were refractive under polarized light, although the level varied (Fig. 2d).

## Conclusions

Histochemical stains and polarized light microscopy demonstrated features characteristic of HRG similar to those reported previously [6, 9, 10, 16, 17]. The omental HRG in this dog was closely associated with the presence of plant materials in the tissue. The hyaline rings were PAS-positive and diastase-resistant, which revealed the cellulose content of HRG, and thus supported its exogenous origin. Plant materials should be distinguished from animal structures that can have morphologic similarities to vegetable cells, e.g. the pericarp can be misdiagnosed as the cuticle of a maggot, the vegetable albumin cells can mimic the fat bodies of the maggot. The analysis under polarised light microscopy is useful in distinguishing plant from animal elements, however some animal structures may show the birefringent properties. On the other hand, the presence of animal structures is mainly related to oral or cutaneous myiasis, but rarely associated with internal infections [18]. Several studies have demonstrated that hyaline rings are residues of plant material and can be of various morphology: roughly ovoid, circular structures; hyaline rod-like shaped structures; hyaline masses lying within the fibrous connective tissue stroma with small calcified basophilic bodies; clearly identifiable plant cells; and metaplastic bone formation [12, 18, 19]. Philipsen and Reichart [2] suggested that ring-like structures were formed during degradation of plant material by host phagocytic cells. Variations in the intensity of PAS and AB stainings might be explained by the loss of mucosubstances in long-lasting granulomas and are potentially related to age of the granulomas. Chronic exposure to inflammation-derived enzymes could probably modify the morphology of the hyaline rings without compromising their antigenicity [20]. Additionally, the plant origin can be of significance as the composition of cellulose depends the most on the age and species of plants [3]. Deposition of collagen and calcification are considered to be a marker of a lesion chronicity. Gueiros et al. [20] demonstrated that more giant cells were present in initial lesions than in older ones with the latter showing small, droplet calcifications within eosinophilic masses. In contrast to cases of human oral HRG, in which thickened hyaline rings could be observed grossly and which had underwent calcification [3], the hyaline structures in the present case were thin and without calcification. Plant material and hyaline structures were weakly positive for Masson trichrome stain as found in other studies [21, 22]. The findings in the present case suggest that the chronic inflammation was mild and the detected lesions were not at an advanced stage.

Omental HRG has only been reported in a woman, where a gastric ulcer with small perforations and passage for food particles into the omental space was suggested as the port of entrance [23]. In the present dog, HRG was an incidental finding and not associated with clinical signs. A follow up carried out 1 year after ovariohysterectomy revealed a normal clinical status. As in humans, uncomplicated HRG seems to have a good prognosis.

HRG should be considered in the differential diagnosis of omental thickening to avoid misdiagnosis and treatment [17, 23]. In our study, the PAS and ZN stainings were used to rule out the presence of fungal, parasitic and mycobacterial organisms, but such organisms were not found. Hence, granulomatous inflammation caused by an infection was excluded as well as the presence of neoplastic lesions. To the best of our knowledge, this is the first published spontaneous case of HRG in an animal.
